# Unveiling economic and humanistic burden of hematologic malignancies in Japan with personal health record data

**DOI:** 10.1038/s41598-026-36287-7

**Published:** 2026-01-27

**Authors:** Saaya Tsutsué, Kenshi Suzuki, Sooyeol Lim, Ryosuke Nishi, Honoka Nakamura, Hiroya Asou, Anila Mathew, Takunari Yoshinaga

**Affiliations:** 1 Gilead Sciences Japan, 1-9-2 Marunouchi, Chiyoda-ku, Tokyo, 100-6616 Japan; 2 Japanese Red Cross Medical Center, Tokyo, Japan; 3 INTAGE Healthcare, Tokyo, Japan

**Keywords:** Productivity loss, Cost, Quality of life, Hematological malignancy, Japan, Database study, Outcomes research, Haematological cancer, Health care economics

## Abstract

To characterize the economic and humanistic burden of patients with hematologic malignancies in Japan using real-world data. Patients with a claim record for a hematologic malignancy who responded to a survey regarding productivity loss and quality of life (QoL) were analyzed to assess their direct medical cost, productivity loss, and QoL along with their levels of comorbidity. This study identified a main cohort of 122 patients with a hematologic malignancy who responded to Work Productivity and Activity Impairment (WPAI) and EQ-5D-5 L questionnaires. Median patient age was 54.5 years (range 25–71 years), with median Charlson Comorbidity Score (CCI) of 2. Most of the mean total cost (USD 12,836.14; JPY 1,670,624) was attributed to productivity loss (mean USD 8,106.39; JPY 1,055,046) rather than direct medical cost (mean USD 4,729.76; JPY 615,578). Presenteeism (mean USD 5,117.84; JPY 666,086) accounted for a greater proportion of productivity loss than absenteeism (mean USD 2,988.55; JPY 388,960). Deterioration in QoL (-0.045) was observed in comparison to the general population in Japan. The results point to the need for a social security policy to provide appropriate support such as better therapies or access for patients who must receive treatment while still working.

**Supplementary Information:**

The online version contains supplementary material available at 10.1038/s41598-026-36287-7.

## Introduction

As society ages, the prevalence of hematologic malignancies is estimated to increase in Japan, with increases in prevalence of + 51.3%, + 28.3%, and + 45.5% for lymphoma, leukemia, and multiple myeloma, respectively, projected by 2050^1^. Such an increase in prevalence of hematological malignancies with reported comorbidities^2^ imply that although patients may live longer with the introduction of innovative therapies^3^, they may also experience impaired physical and emotional states for a sustained period of time^4^. This can have a large potential impact on patients’ labor productivity and quality of life (QoL), which societal level support may need to address^5^.

While several previous studies have reported on direct medical cost for patients with hematologic malignancies in Japan^6–11^, direct medical cost may be only one of the elements of the overall burden of illness^12^. A study of lymphoma patients in the US^13^ showed the indirect cost of treatment of lymphoma to be substantial; for example, the mean estimated cost of absenteeism for a patient with mantle cell lymphoma was USD 1,606 per patient per month in 2019. Another study also demonstrated the presence of a sizable amount of productivity loss for patients with diffuse large B-cell lymphoma^14^. This suggests that productivity loss may need to be regarded as a pressing issue from the societal perspective, through which the full economic burden of a disease may be more fully appreciated. From a policy standpoint, a societal view might show that a treatment’s high efficacy may allow patients and their caregivers to return to work, making it more cost-effective for the national economy than a payer-only perspective would indicate. To our knowledge no data has been reported on productivity loss caused by hematological malignancies in Japan.

QoL is also meaningful from the individual patient perspective as it measures patients’ unique experiences of the impact of the disease on their well-being along physical, psychological, and social dimensions^15^, the magnitude of which may be difficult to assess based on conventional secondary databases alone without patient reported outcomes (PRO). Thus, QoL also needs to be considered as an additional factor for the comprehensive assessment of the burden of illness for hematologic malignancies in Japanese the real-world setting.

Although there have been previous studies on indirect cost and employment^16,17^ and QoL^16,18,19^ for patients with hematologic malignancies, to our knowledge, this study is the first to use personal health records (PHR) collected by an app on hand-held devices linked to a health insurance claims database to investigate the overall burden of illness in hematologic malignancies, with the claims database also supplying information from survey non-responders for comparative assessment of findings from survey responders. Such information can be useful in gaining a fuller appreciation of the value of innovative therapies as shown by HTA reviews in Sweden^20^ and Denmark^21^. Thus, this study aims to quantify the social cost of hematologic malignancies in Japan. Such analysis can potentially aid assessment of the value of innovative therapies which is under healthcare policy discussion in Japan.

## Methods

This study is a retrospective cohort database study utilizing the Japan Medical Data Center (JMDC) database. The JMDC database is an electronic health records-based database which contains anonymized insurance claims data from health insurance associations in Japan, including data for 20 million people who are either employed full-time or dependent family members of fully employed members^22^. This study also retrospectively utilized the survey response data delivered by a novel technology associated with the JMDC database called Pep Up that allows deterministic linkage between survey responses and claims data for the same survey respondents using a unique, anonymized patient identifier^23^ permitting integration of claims data with PRO. The integrated data was used to evaluate the economic burden in terms of direct medical cost and productivity loss as well as the humanistic burden in terms of QoL derived from past questionnaire responses for patients with hematologic malignancies of interest, along with their demographic characteristics and comorbidities determined from the claims data. Due to the employment-based nature of the database, the JMDC database has an age distribution representing the working-age population of Japan, which is suitable for the objective of this study that aims to investigate productivity loss. The database includes both inpatient and outpatient claims data, with information on diagnoses, drugs prescribed, procedures and laboratory tests performed.

**Fig. 1:**
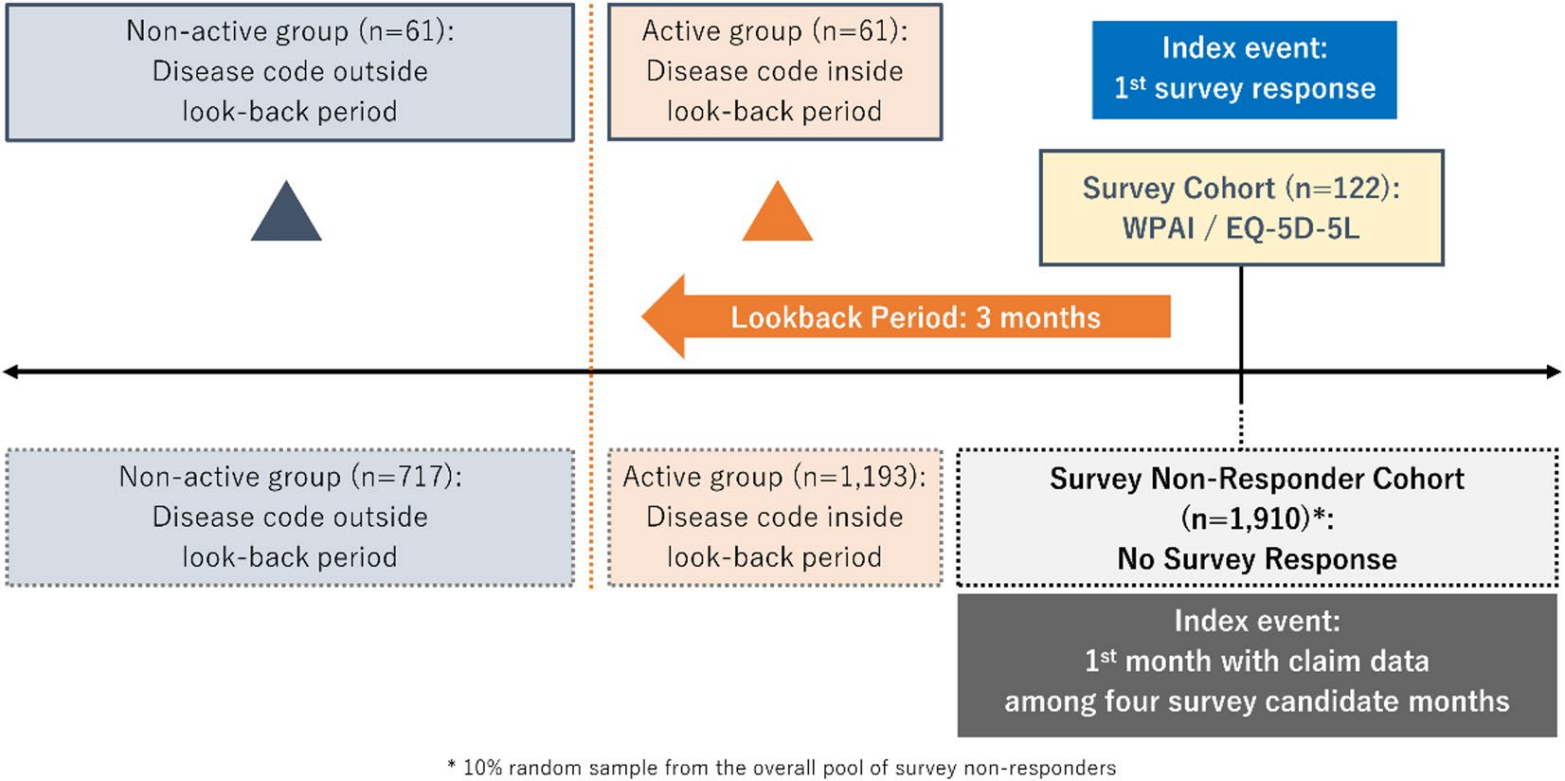
Definition of main and exploratory cohorts.

The identification period of this study was from January 2015 until June 2022. The patients of main interest (Fig. 1) were those who responded to at least one of the four past surveys conducted through Pep Up in February, June, December 2021, and June 2022, the timing of which was determined by JMDC and was not related to any specific clinical events. The index event was defined as the first survey response, and the index month was defined as the month of the index event as the survey timing was recorded only in monthly units within the JMDC database. In order to be included within the main cohort, patients had to be aged ≥ 18 years at the index month, had at least one confirmed disease code for one of the target hematologic malignancies such as diffuse large B-cell lymphoma (DLBCL), mantle cell lymphoma (MCL), follicular lymphoma (FL), adult T-Cell leukemia lymphoma (ATLL), acute lymphoblastic leukemia (ALL), acute myeloid leukemia (AML), multiple myeloma (MM), myelodysplastic syndrome (MDS), and other non-major hematologic malignancies (full code list given within Supplementary Table 1) any time prior to and including the index month, and meeting the requirement for a look-back period of 3 months of continuous enrollment including the index month. To aid the assessment of internal consistency of findings from the main cohort, an exploratory cohort of survey non-responders was defined based on a 10% random sample of the survey non-responders to mitigate computational burden^24^ (Fig. 1), with cohort selection criteria similar to the main cohort except that the index event was defined as the first month with a claim among the four candidate survey months. Consolidated Standards of Reporting Trials (CONSORT) diagrams are available within the supplementary material for visual demonstration of the selection criteria for the main and exploratory cohorts (Supplementary Fig. 1 A and 1B). Within each of the main cohort and the exploratory cohort, sub-cohorts were defined by the status of hematologic malignancies (Fig. 1), with the active group consisting of patients with at least one claim of hematologic malignancies within the 3 months look-back period, and the non-active group consisting of patients without any claim of hematologic malignancies within the look-back period but with at least one claim of hematologic malignancies before the look-back period. For hematological malignancies, follow-up visit frequencies may decrease to every 3 to 6 month intervals several years after treatment is completed^25^. Therefore, the length of the 3 months of look-back period was deemed reasonable to classify patients according to the level of recent disease activity around the time of survey. The presence of recent disease activity was hypothesized to lead to greater direct cost outcomes and productivity loss as well as lower QoL.

### Ethics approval and consent to participate

This study retrospectively utilized an existing health insurance claims database, along with previously accumulated PRO data for patients who consented to participate in the surveys deployed through Pep Up by JMDC. All data in this database were deidentified, and patient records were only linked within each health insurance plan. As such, because of anonymization of the data, the Ethical Guidelines for Epidemiological Research in Japan are not applicable to this study^26^. In addition, the Ethical Guidelines on Biomedical Research Involving Human Subjects ascertain that written informed consent from patients is not required for such pharmaco-epidemiological studies conducted using medical databases, as the use of pre-existing data does not require any interaction with patients^27^. However, to ensure an objective evaluation of the ethical standard followed in this research through an optional third-party review, an IRB review was conducted. This non-interventional study was approved by the Ethics Committee of the Medical Corporation TOUKEIKAI Kitamachi Clinic Ethical Review Board on September 18, 2024 (Approval number: KXJ10419). All procedures were conducted in accordance with the Declaration of Helsinki and the Japanese Ethical Guidelines for Medical and Health Research Involving Human Subjects and its guidance document.

### Data analysis

The following outcomes were collected and analyzed in this study: patient characteristics, productivity loss from WPAI^28^, direct medical cost and healthcare resource utilization (HCRU) during the look-back period from claims data for economic outcomes, QoL scores from EQ-5D-5 L based on the Japanese version of the value set^29,30^ and the activity impairment score from WPAI for humanistic outcomes, and comorbidities of interest including history of other malignancies and Charlson Comorbidity Index (CCI) with a modification by Deyo et al.^31,32^ during the look-back period for clinical outcomes. Activity impairment score comes from WPAI but in this study it does not impact the productivity loss calculation; therefore, it was placed under humanistic outcomes. For calculation of the direct medical cost, the unit cost information from the Japanese reimbursement table was applied. Cost items were also categorized into various groups such as inpatient cost, outpatient cost, cancer treatment cost, bleeding treatment cost, infection treatment cost, cost related to laboratory tests, pharmacy cost, radiation therapy cost, and stem cell transplantation (SCT) cost.

For calculation of productivity loss (annualized indirect cost) from WPAI in terms of Japanese yen (JPY)^33^, the calculation was carried out as below, using the human capital approach:

Total Cost of Lost Productivity = Cost of Absenteeism + Cost of Presenteeism = [(Absenteeism defined as hours of missed work per week due to health issues) x (Average hourly wage) x 50 work weeks] + [((Hours of actual work per week) x (Presenteeism defined as health impact on productivity while working on a scale of 0–10)/10) x (Average hourly wage) x 50 work weeks]

This study adheres to the recommendations of the “Guidelines for Cost-Effectiveness Evaluation Analysis” issued by C2H^34^ and endorsed by the Central Social Insurance Medical Council of Japan. This guideline stipulate that cost estimation should employ data from the most recent point in time possible. In line with this principle, the present study has taken the following approach:

The hourly wage used was the population mean derived as the average for all industries, all ages, and all genders, based on the latest Basic Survey on Wage Structure in 2023^35^ per recommendation by C2H in Japan^34^.

Direct medical cost outcomes were adjusted to the most recent year of 2024 with available data at the time of analysis per recommendation by C2H^34^ by the medical price index in Japan per earlier studies^7,9^ (list of price index given in Supplementary Table 2) which reflects annual adjustment by the Japanese government in various medical costs and fees subject to reimbursement. Costs were first calculated in Japanese Yen (JPY) and presented in US dollars (USD) after adjustment by the aforementioned medical price index and through foreign exchange conversion at the rate (1 USD = 130.15 JPY) at the end of January 2023 published by the Bank of Japan^36^, to be consistent with the year from which the hourly wage statistic for productivity loss was taken.

HCRU measures included frequencies of outpatient visits, intensive care unit (ICU) admissions, high care unit (HCU) admissions, and total length of stay. HCRU and costs were reported on a per patient per year basis after annualization of the estimates obtained during the look-back period for ease of interpretation^37^.

For multivariate statistical modeling, fractional logistic regression models^38^ were used for outcomes with bounded ranges such as the WPAI overall impairment score and the QoL scores; a generalized linear model (GLM) with a Tweedie distribution^39^ which can handle a continuous non-negative dependent variable was used for direct medical cost in order to be able to handle patient data with zero direct medical cost recorded during the look-back period; the logistic regression model was used to model non-working status. The covariates used for multivariate statistical modeling were age, gender, CCI, and the presence of recent disease activity (a disease code for a hematologic malignancy) during the look-back period. Statistical significance of covariates was determined in a conventional manner with p-values associated with each parameter estimate. In addition, marginal effects were also estimated as a practical aid for interpretation of covariate effect estimates from various GLM-based regression modeling results^40^. For tabulations and main statistical analyses, observed cases without missing data were used. Sensitivity analyses were also conducted to assess the impact of missing data for WPAI and QoL through imputation via predictive mean matching.

QoL scores were also compared among the three groups: the employed survey responders with WPAI overall impairment score < 20, the employed survey responders with WPAI overall impairment score ≥ 20, and the unemployed survey responders following a similar approach to an earlier study in breast cancer^41^. An analysis of variance (ANOVA) as well as pairwise t-tests were conducted to compare QoL scores among those three groups. Disutility associated with hematologic malignancies (reduction in QoL in comparison to the Japanese population norm) was estimated by calculating differences from population norm values of EQ-5D-5L among various gender and age categories of the general population sample in Japan reported in an earlier study^42^. Although activity impairment score is a part of the WPAI questionnaire and measures productivity impacts outside of paid employment, it does not capture productivity within the sphere of the workplace; thus, its results were presented together with QoL measures in this study. Pearson correlations were calculated with confidence intervals (CI) and p-values from pairwise tests among overall impairment scores of WPAI, QoL scores, and direct medical cost for patients. The direct cost and comorbidities of the exploratory cohort of survey non-responders were also calculated for assessment of internal consistency of results from the main cohort. The data for the main cohort were also checked as exploratory analyses for the presence of the following records throughout the enrollment period up to the time of survey: (1) issuance of a medical opinion letter for injury and illness benefits (Japanese medical fee code: B012) as another source of evidence for absenteeism, (2) chimeric antigen receptor T cell (CAR-T) therapies available during the study period in Japan (Japanese *receiptcode* 629700401, 629700601, 629700701, 629701001). For exploratory assessment of potential impact of COVID-19, the presence of a diagnosis of COVID-19 (ICD-10 code: U071) was also confirmed as supplementary information.

All data analyses were performed in R version 4.2 or higher. For descriptive statistics, continuous study measures were summarized as means, standard deviations (SD), medians (with interquartile ranges [IQR]), minimum and maximum values. Categorical variables were described by reporting frequencies (N) and percentages (%). All CIs were estimated as two-sided 95% CI, and statistical significance was assessed at the alpha level of 0.05. T-test for continuous variables and Fisher’s exact test for categorical variables were performed. All analyses were performed in a manner consistent with the Strengthening the Reporting of Observational Studies in Epidemiology (STROBE) guidelines^43^.

## Results

### Patient characteristics

During the study identification period (January 2015 until June 2022), there were 38,923 patients with a diagnosis code for one of the hematologic malignancies of interest. After application of inclusion and exclusion criteria, there were 122 patients in the main cohort of survey responders and 1,910 patients in the exploratory cohort of survey non-responders. The patient selection process is illustrated in the CONSORT diagrams of Supplementary Fig. 1 A & B. Within the main cohort, there were 61 patients in the active group and 61 patients in the non-active group; within the exploratory cohort, there were 1,193 patients in the active group and 717 patients in the non-active group (Fig. 1).

Patient demographics of the main cohort (Table 1, Supplementary Table 3) show that the mean (SD; median) age of the main cohort is 52.3 (9.6; 54.5) years. 70 patients (57.4%) were male, and 52 patients (42.6%) were female. There were no statistically significant differences in age and gender between the active and non-active groups. The most commonly identified hematologic malignancies in the main cohort included non-Hodgkin lymphoma, unspecified (*n* = 69, 56.6%), multiple myeloma (MM) (*n* = 17, 13.9%), myelodysplastic syndromes (MDS) (*n* = 14, 11.5%), diffuse large B-cell lymphoma (DLBCL) (*n* = 12, 9.8%), and acute myeloid leukemia (AML) (*n* = 12, 9.8%) (Supplementary Table 1).

**Table 1:** Patient baseline characteristics and comorbidities.

	Total	Active	Non-active	*p*-value^1^
	(*n* = 122)	(*n* = 61)	(*n* = 61)	
**Age at index month (years)** :				0.380
Mean (SD)	52.3 (9.6)	51.5 (9.9)	53.0 (9.2)	
Median (Q1, Q3)	54.5 (47, 59)	53 (48, 59)	55 (47, 59)	
Min, Max	25, 71	25, 68	29, 71	
**Gender**,** n(%)** :				0.360
Male	70 (57.4%)	38 (62.3%)	32 (52.5%)	
Female	52 (42.6%)	23 (37.7%)	29 (47.5%)	
**Comorbidities**,** n(%)** :				
Chronic hepatitis	10 (8.2%)	8 (13.1%)	2 (3.3%)	0.095
Congestive Heart failure	9 (7.4%)	7 (11.5%)	2 (3.3%)	0.163
Kidney disease	10 (8.2%)	6 (9.8%)	4 (6.6%)	0.743
Liver disease	19 (15.6%)	13 (21.3%)	6 (9.8%)	0.133
Hypertension	24 (19.7%)	15 (24.6%)	9 (14.8%)	0.255
Lymph nodes enlarged	1 (0.8%)	1 (1.6%)	0 (0.0%)	1.000
Chronic pulmonary disease	25 (20.5%)	20 (32.8%)	5 (8.2%)	0.001*
Rheumatic disease	8 (6.6%)	4 (6.6%)	4 (6.6%)	1.000
Diabetes with complications	4 (3.3%)	4 (6.6%)	0 (0.0%)	0.119
History of other malignancy**	63 (51.6%)	55 (90.2%)	8 (13.1%)	< 0.001*
Myelodysplastic syndromes (MDS)	8 (6.6%)	8 (13.1%)	0 (0.0%)	0.006*
**CCI score** :				< 0.001*
Mean (SD)	2.1 (2.1)	3.4 (1.8)	0.9 (1.6)	
Median (Q1, Q3)	2 (0, 3)	3 (2, 4)	0 (0, 1)	
Min, Max	0, 9	0, 8	0, 9	

1 : t-test for continuous variables; Fisher’s exact test for categorical variables.

*p < 0.05 : statistical significance.

**Consistent with the CCI definition, including lymphoma and leukemia but not MDS or metastatic solid tumor.

Patient comorbidities during the look-back period of the main cohort (Table 1) show that the mean (SD; median) CCI score was 2.1 (2.1; 2) for the main cohort, and the active group had a statistically significantly greater CCI (mean: 3.4; SD: 1.8; median: 3) than the non-active group (mean: 0.9; SD: 1.6; median: 0) (*p* < 0.001). Disease codes for non-oncological comorbidities such as chronic pulmonary diseases (20.5%), hypertension (19.7%), liver diseases (15.6%), chronic hepatitis (8.2%), kidney diseases (8.2%), congestive heart failure (CHF) (7.4%) and rheumatic diseases (6.6%) were found for > 5% of the patients of the main cohort during the look-back period.

Patient demographics and comorbidities of the exploratory cohort of survey non-responders (Supplementary Table 4) show that the exploratory cohort of survey non-responders had a similar age (mean: 51.7; SD: 12.8; median: 54) yet a slightly higher level of comorbidity by CCI (mean: 2.7; SD: 2.5; median: 2) in comparison to the main cohort, suggesting that survey responders may have been slightly healthier than survey non-responders.

Within the main cohort, there were no CAR-T therapy recipients, and few patients had a clear diagnosis record of COVID-19 (Supplementary Table 5).

### Economic and humanistic outcomes

#### Total cost

The mean USD (JPY) total cost per year for patients with hematologic malignancies for the main cohort (Table 2 for USD; Supplementary Table 6 for JPY) was USD 12,836.14 (JPY 1,670,624) per year, with the productivity loss of USD 8,106.39 (JPY 1,055,046) per year accounting for a greater proportion (63%) of the total cost than the direct medical cost of USD 4,729.76 (JPY 615,578) per year.

**Table 2:** Total cost, direct medical cost, productivity loss (USD).

	Total	Active	Non-active	*p*-value^1^
	(*n* = 122)	(*n* = 61)	(*n* = 61)	
**Total cost (USD)** :				0.040*
N Non-missing	99	52	47	
Mean (SD)	12,836.14 (24,792.13)	17,557.30 (30,362.66)	7,612.73 (15,306.90)	
Median (Q1, Q3)	3,965.04 (665.24, 14,735.88)	7,338.10 (1,675.69, 22,972.73)	1,794.89 (325.61, 8,368.54)	
Min, Max	0, 159,597.06	243.05, 159,597.06	0, 85,602.94	
Total direct medical cost** (USD) :				0.130
N Non-missing	99	52	47	
Mean (SD)	4,729.76 (13,429.58)	6,638.96 (15,614.25)	2,617.45 (10,258.99)	
Median (Q1, Q3)	1,396.34 (385.14, 3,222.35)	1,836.52 (893.44, 4,383.68)	658.75 (205.37, 1,947.66)	
Min, Max	0, 96,072.63	223.42, 96,072.63	0, 71,014.76	
**Work productivity loss - overall (USD)** :				0.095
N Non-missing	99	52	47	
Mean (SD)	8,106.39 (18,193.35)	10,918.34 (23,114.72)	4,995.29 (9,694.15)	
Median (Q1, Q3)	0 (0, 9,117.61)	3,874.99 (0, 13,129.36)	0 (0, 8,205.85)	
Min, Max	0, 152,264.13	0, 152,264.13	0, 49,782.17	

USD amounts are in annualized units (USD per year).

1 : t-test.

*p < 0.05 : statistical significance.

**For patients who have non-missing values of work productivity loss. Work productivity loss is expressed in non-missing monetary quantity only when patients can have valid monetary conversion for all values of absenteeism, presenteeism, and overall productivity loss.

#### Productivity loss

The mean USD (JPY) productivity loss per year for the main cohort was USD 8,106.39 (JPY 1,055,046) per year, with presenteeism of USD 5,117.84 (JPY 666,086) per year accounting for a greater proportion (63%) of productivity loss than absenteeism of USD 2,988.55 (JPY 388,960) per year (Supplementary Table 7 for USD; Supplementary Table 8 for JPY).

The fractional regression model of productivity loss (Supplementary Table 9 A) shows that.

higher age was statistically significantly associated with a lower overall impairment score (*p* = 0.001). The sensitivity analysis with imputation of missing data (Supplementary Table 9B) also showed consistent results.

The majority of the survey respondents (86.1%) were employed (Supplementary Table 7). The logistic regression model of non-working status (Supplementary Table 10) shows that higher age (*p* = 0.003) and female gender (*p* = 0.002) were statistically significantly associated with non-working status. Among the main cohort, 14 patients (11.5%) received a medical opinion letter for injury and illness benefits throughout the enrollment period.

#### QoL measures: EQ-5D-5L and activity impairment score

The main cohort reported the mean QoL of 0.890 (SD: 0.148; median: 0.895) based on EQ-5D-5 L (Supplementary Table 11). Upon comparison with the QoL values of the sample of the general population in Japan reported by Shiroiwa et al.^42^, the reported QoL yielded the mean disutility (loss in QoL) of −0.045 (SD: 0.149; median: −0.030), which was statistically significantly different from the disutility of 0 (*p* = 0.002) (Table 3).

**Table 3:** Disutility of hematologic malignancies.

	Total	Active	Non-active	*p*-value^2^
	(*n* = 122)	(*n* = 61)	(*n* = 61)	
**Disutility estimate**				0.486
N Non-missing	116	58	58	
Mean (SD)	−0.045 (0.149)	−0.055 (0.160)	−0.035 (0.138)	
Median (Q1, Q3)	−0.030 (−0.108, 0.069)	−0.030 (−0.116, 0.069)	0.047 (−0.100, 0.070)	
Min, Max	−0.830, 0.075	−0.830, 0.075	−0.477, 0.075	
p-value^1^	0.002*	0.012*	0.056	

1 : One-sample t-test of disutility for the corresponding cohort.

2 : Two-sample t-test between the active and non-active sub-cohorts.

*p < 0.05 : statistical significance.

The fractional regression model of QoL scores (Supplementary Table 12 A) shows that.

higher age (*p* = 0.007) and lower CCI (*p* = 0.003) were statistically significantly associated with higher QoL scores. The sensitivity analysis with imputation of missing data (Supplementary Table 12B) also showed consistent results.

As for the activity impairment score from WPAI, there was a statistically significant difference between the active group (mean: 22.8; SD 29.8; median: 5) and the non-active group (mean: 12.1; SD: 21.4; median: 0) (Supplementary Table 7). The fractional regression model of activity impairment scores (Supplementary Table 13) showed that lower age (*p* < 0.001) and female gender (*p* = 0.042) were statistically significantly associated with higher activity impairment scores.

#### Relationship among productivity loss, QoL, and direct medical cost

**Fig. 2:**
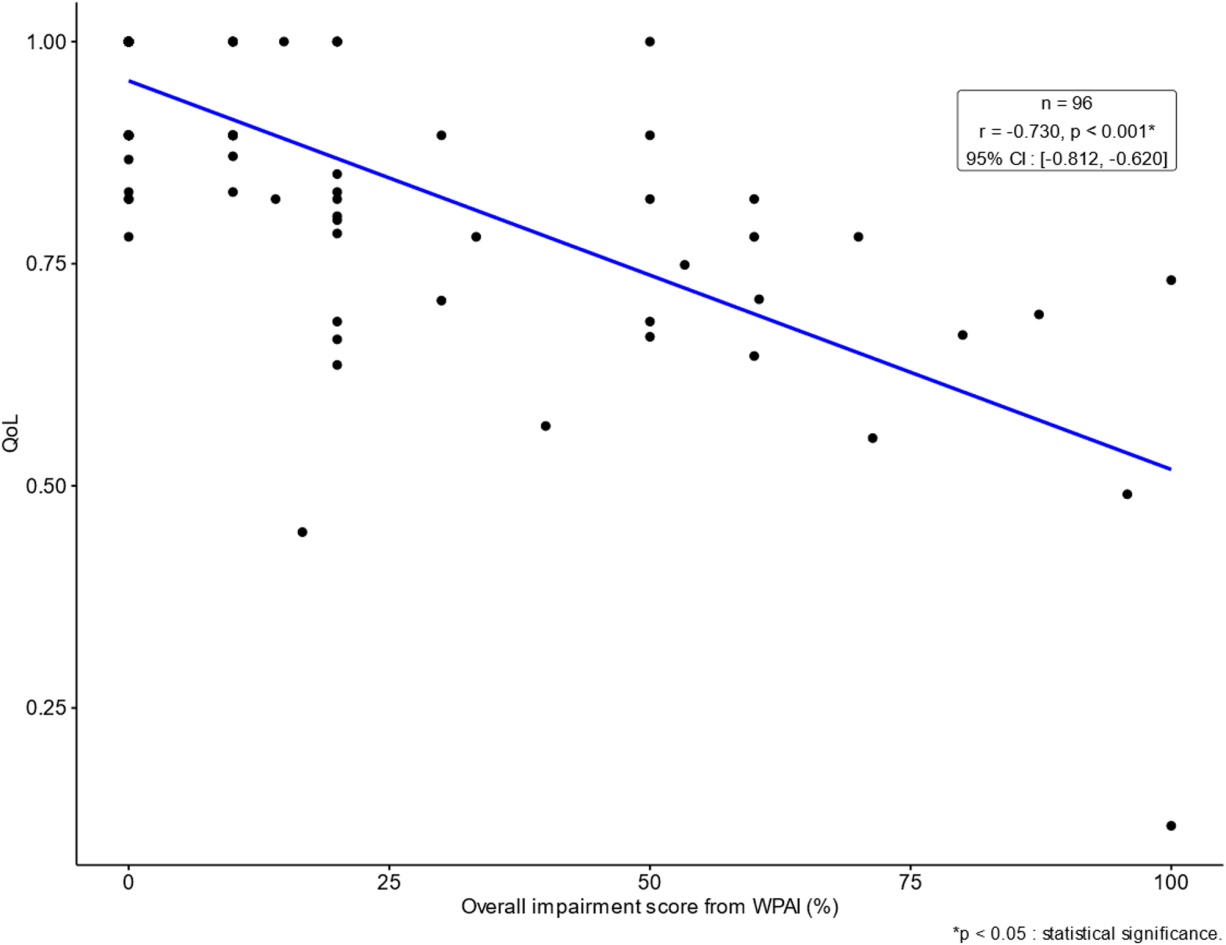
Scatterplot and correlation between productivity loss and QoL.

There was a statistically significant strong negative correlation between productivity loss and QoL scores (*r* = −0.730; *p* < 0.001) (Fig. 2). There was a statistically significant moderate positive correlation between productivity loss and direct medical cost (*r* = 0.277; *p* = 0.006). There was a statistically significant negative correlation between QoL and direct medical cost (*r* = −0.212; *p* = 0.022).

#### Relationship among employment, presenteeism and QoL

**Fig. 3:**
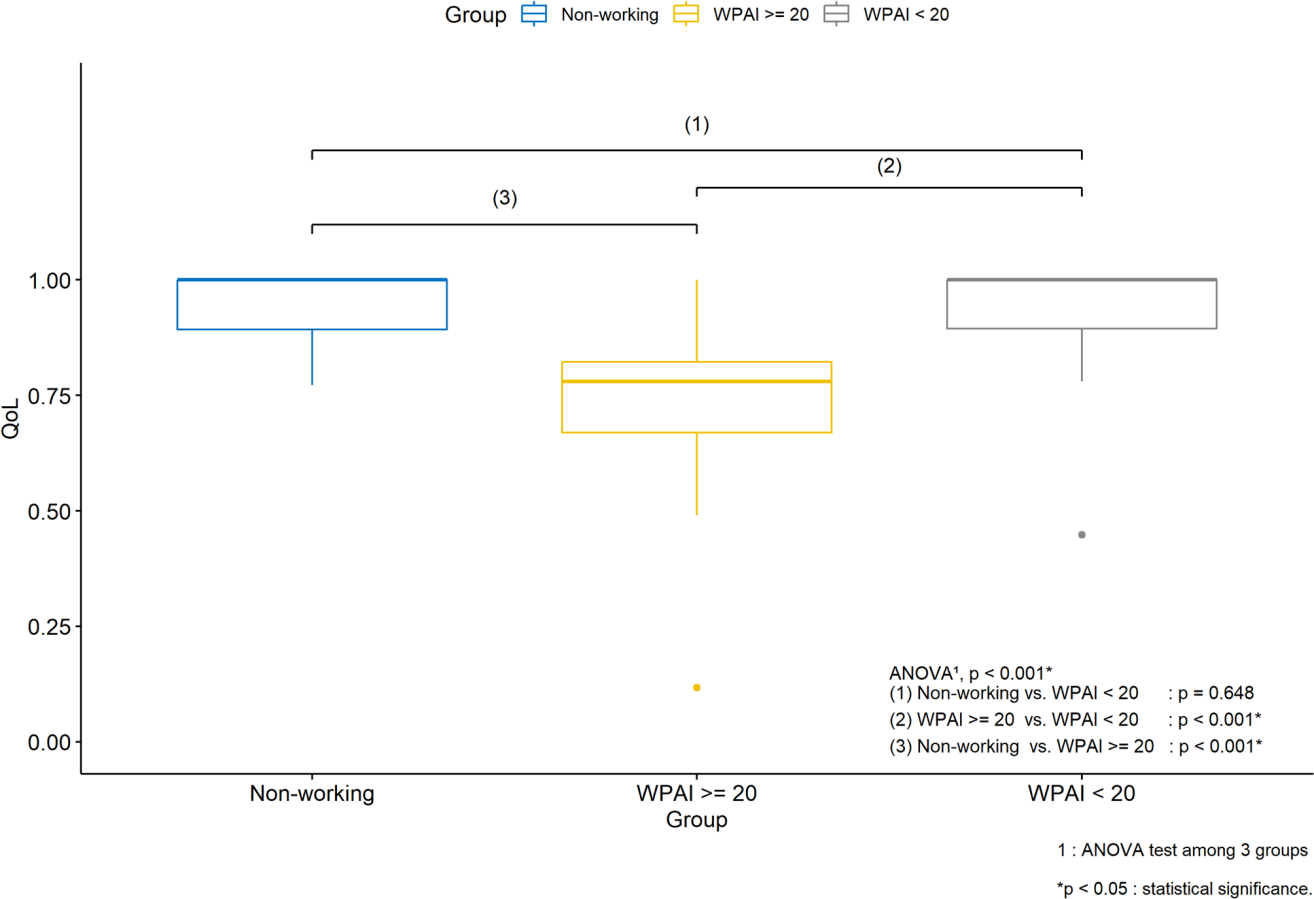
Boxplot of QoL scores among categories defined by employment status and WPAI scores.

Employed survey respondents with a relatively large productivity loss (WPAI overall impairment score ≥ 20) had the lowest mean QoL of 0.747 (SD: 0.171; median: 0.780) than either the unemployed survey respondents (QoL mean: 0.939; SD: 0.077; median: 1) or the employed survey respondents with a relatively small productivity loss (WPAI overall impairment score < 20) (QoL mean: 0.950; SD: 0.091; median: 1) (*p* < 0.001) (Table 4; Fig. 3). On the other hand, the difference in QoL between the unemployed survey respondents and the employed survey respondents with a relatively small productivity loss was not statistically significant (*p* = 0.648).

**Table 4:** Analysis of QoL by employment status and WPAI categories.

			Working		
	Total	Not working	WPAI > = 20	WPAI < 20	*p*-value^1^
	(*n* = 115)	(*n* = 17)	(*n* = 33)	(*n* = 65)	
**Utility weighted by the Japanese tariff** :					< 0.001*
N Non-missing	112	16	33	63	
Mean (SD)	0.888 (0.149)	0.939 (0.077)	0.747 (0.171)	0.950 (0.091)	
Median (Q1, Q3)	0.895 (0.823, 1)	1 (0.892, 1)	0.780 (0.670, 0.823)	1 (0.895, 1)	
Min, Max	0.117, 1	0.772, 1	0.117, 1	0.448, 1	

1 : ANOVA test among 3 groups.

*p < 0.05 : statistical significance.

#### Direct medical cost & HCRU

The mean total direct medical cost was USD 5,543.83 (JPY 721,529) per year among all patients (*n* = 122) in the main cohort (Supplementary Table 14 A, 14B), whereas it was USD 4,729.76 (JPY 615,578) per year among those with both non-missing total direct cost and productivity loss (*n* = 99) (Table 2, Supplementary Table 6). The mean inpatient cost of USD 2,321.99 (JPY 302,206) per year was greater than the mean outpatient cost of USD 2,070.32 (JPY 269,452) per year or the mean pharmacy cost of USD 1,151.53 (JPY 149,871) per year (Supplementary Table 14 A, 14B). Regarding other annualized cost items for patients in the main cohort, the mean lab test cost was USD 1,352.83 (JPY 176,070), the mean cancer treatment cost was USD 980.42 (JPY 127,601), the mean bleeding treatment cost was USD 411.73 (JPY 53,587), the mean infection treatment cost was USD 197.94 (JPY 25,762), and the mean radiation therapy cost was USD 155.05 (JPY 20,180).

Annualized outpatient visits (Supplementary Table 15) were mean 15.3 times (SD: 14.1; median: 8) per year and annualized total length of stay was mean 7.1 days (SD: 41.6; median: 0) per year. No ICU or HCU admissions were found for the main cohort.

The GLM with a Tweedie distribution for direct medical cost of the main cohort (Supplementary Table 16 A, 16B) showed that higher CCI (*p* = 0.037) and recent disease activity for a hematologic malignancy (*p* = 0.036) were statistically significantly associated with higher direct medical cost.

#### Impact of disease activity level on total cost, productivity loss and QoL

The total cost for the active group was USD 17,557.30 (JPY 2,285,083) and the total cost of for the non-active group was USD 7,612.73 (JPY 990,797), resulting in a statistically significant difference (*p* = 0.040) (Table 2, Supplementary Table 6).

The presenteeism cost for the active group was USD 6,657.61 (JPY 866,488) and the presenteeism cost of for the non-active group was USD 3,414.25 (JPY 444,365), resulting in a statistically significant difference (*p* = 0.048) (Supplementary Tables 7, 8). The proportion of patients with a record for issuance of a medical opinion letter for injury and illness benefit throughout the enrollment period showed a statistically significant difference (*p* = 0.044) between the active group (*n* = 11; 18.0%) and the non-active group (*n* = 3; 4.9%).

Regarding individual components of the direct medical cost, there were statistically significant differences in cancer treatment cost (*p* = 0.036), laboratory test cost (*p* = 0.004), outpatient visit cost (*p* = 0.015) between the active group and the non-active group (Supplementary Table 14 A, 14B). The GLM model also shows that after adjusting for the impact of covariates, the non-active group incurred USD 5,004.28 (JPY 649,795) less direct medical cost than the active group on average (*p* = 0.035; Supplementary Table 16 A, 16B).

Regarding QoL, there was no statistically significant difference (*p* = 0.486) in disutility between the active group (mean: −0.055; SD: 0.160; median: −0.030) and the non-active group (mean: −0.035; SD: 0.138; median: 0.047) (Table 3).

#### Cost and HCRU outcomes from the exploratory cohort of survey non-responders

The exploratory cohort of survey non-responders tended to have higher direct medical cost outcomes (Supplementary Table 17 A, 17B) and HCRU outcomes (Supplementary Table 18) than the main cohort of survey responders (Supplementary Table 14 A, 14B, 15). The GLM with a Tweedie distribution for direct medical cost of the exploratory cohort (Supplementary Table 19 A, 19B) showed a similar trend as the result of the main cohort (Supplementary Table 16 A, 16B).

## Discussion

This study is the first to comprehensively evaluate productivity loss and QoL from patient responses to a survey as well as direct medical cost and clinical comorbidity levels determined from a secondary database for the same group of patients with hematologic malignancies in Japan. Results indicate that productivity loss may be a hidden driver of social cost with potential implications for the social value of therapies and the appropriate level of pricing in the era of innovative regenerative medicine^44^.

A notable aspect of the this study is that it allows further insight into the relationship between clinical parameters and real-world outcomes by supplying information that is not typically available through conventional randomized clinical trials (RCT)^45,46^. One of the examples demonstrated in this study is the statistically significant impact of increasing CCI on direct medical cost and QoL. The marginal effect estimates in the statistical models imply that increasing CCI by one unit may lead to approximately USD 1437.24 (JPY 187,722) increase in direct medical cost per year (Supplementary Table 16 A, 16B) and − 0.015 reduction in EQ-5D-5L score (Supplementary Table 12 A) on average among survey responders in the real-world settings. This result is consistent with the earlier research findings that QoL was lower among DLBCL patients with greater comorbidity^19^ and financial burden was associated with lower QoL among patients with hematologic malignancies^45^.

This study showed the presence of a sizable amount of productivity loss (mean USD 8,106.39 per year; JPY 1,055,046) that was greater (63% of the total cost) than the annualized direct medical cost (mean USD 4,729.76 per year; JPY 615,578) among the patients of hematologic malignancies who responded to a survey (Table 2, Supplementary Table 6), even though a half of the survey responders were in a state of low disease activity close to the time of survey. The annualized cost of presenteeism for hematologic malignancies (mean USD 5,117.84; JPY 666,086) per patient in Japan was also notable (Supplementary Tables 7, 8). The relative impact of productivity loss in comparison to direct medical cost can be a benchmark for formulating public policy for regenerative medicine which needs to assess the full extent of the socioeconomic impact of a disease or an intervention for optimal allocation of budgetary resources^47^. From this perspective, a potentially curative treatment option could mitigate disease burden across a longer-term horizon and thus result in considerable cumulative social benefit. This suggests that a sizable proportion of patients with hematologic malignancies in Japan may be able to mitigate or avoid productivity loss across multiple years if they can access a potentially curative therapy.

At the same time, trends such as the group in active treatment exhibiting greater levels of comorbidities and various direct cost outcomes than the non-active group were relatively similar between the main cohort of survey responders and the exploratory cohort of survey non-responders, supporting internal consistency of results from the survey responders. General directional trends of outcomes such as the strong negative relationship between productivity loss and QoL, more disease activity corresponding to higher cost outcomes, and the negative disutility of hematologic malignancies in comparison to the general population^19,48^.

The advantage of conducting a hybrid study design which combined the use of PRO and a secondary database also illustrates the benefits of this study design as opposed to doing two separate studies with different patient samples. As one example, for investigation of absenteeism, the analysis of patient reported WPAI can be complemented by the claims data analysis of a medical opinion letter for injury and illness benefits which showed that 11.5% of survey responders may have suffered a high degree of absenteeism as medical leave from work in the past. This finding from the claims data hints at presence of a high degree of absenteeism among some patients in the long term that was not accounted for by the WPAI responses focusing on survey respondents’ short-term experiences. Thus, collective financial impact of absenteeism across progression of hematologic malignancies may be greater than the estimate of USD 2,988.55 per year (JPY 388,960) derived from the WPAI responses only (Supplementary Tables 7, 8). As another example, examination of correlations among productivity loss, QoL and direct medical cost showed that unlike the strong correlation (*r* = −0.730) between productivity loss and QoL, the impact of direct medical cost estimated from the claims data on patient-reported productivity loss (*r* = 0.277) and QoL (*r* = −0.212) could be potential future work to clarify in prospective research format. As a third example, comparison of the effect of age on outcomes suggests that while age had relatively limited effect on direct cost once adjusted for the effect of other covariates such as CCI and recent disease activity, a higher age was statistically significantly associated with a higher QoL and a lower productivity loss. This observation could be potentially explained by the finding from a systematic review that younger age was a risk factor for financial toxicity in hematologic malignancies^49^ and the report that the degree of presenteeism in young to middle-aged workers tended to be higher in general, possibly because they may feel under pressure to attend work even when they feel unwell due to career-related concerns^50^. It should also be noted that some patients in advanced age may have chosen to retire and drop out of the workforce, thereby excluding them from the productivity loss calculation. Similarly, those patients over the retirement age would be excluded from the QoL questionnaire linked to the employment-based insurance claims database. Thus, findings of this study should be interpreted in the context of patients with hematological malignancies within working ages.

This study also fills in the evidence gap regarding the negative impact of hematologic malignancies on QoL in comparison to the general population in Japan. EQ-5D-5 L score is particularly low among working patients with a high level of productivity loss (mean 0.747) in comparison to the employed survey responders with a low level of productivity loss (mean 0.950) and the unemployed survey responders (mean 0.939) (Table 4), consistent with the finding from an earlier study in a solid tumor^41^. This highlights potential need for care and supports directed towards patients who must continue to work for economic reasons even when they feel debilitated by the conditions induced by hematological malignancies. It is also interesting to note that the disutility of −0.045 estimated for hematologic malignancies is similar in magnitude to the reported Minimally Important Difference (MID) of EQ-5D-5 L in adults with Type 2 diabetes ranging from 0.034 to 0.049^51^. MID of EQ-5D-5 L may vary with treatment type^52^, so another potential direction of future research is to investigate what the appropriate level of MID would be for patients eligible for innovative cell therapies in Japan.

As for the hypothesis that the disease activity level of hematologic malignancies may increase direct cost outcomes and productivity loss while reducing QoL, this was directionally consistent with tabulation of observed results. However, the trend was stronger for cost and productivity measures; statistically significant differences were demonstrated between the active and non-active groups for total cost, presenteeism and issuance of a medical opinion letter for injury and illness benefit which served as the proxy measure of a high degree of absenteeism in the past; the impact on direct medical cost was also statistically significant after adjusting for the impact of covariates in a statistical model. However, as for QoL measures, although a statistically significant difference was observed between the two groups for tabulation of activity impairment score from WPAI, there was no statistically significant difference in disutility in terms of EQ-5D-5L. Because EQ-5D-5L is a composite measure consisting of multiple dimensions (mobility, self-care, usual activities, pain/discomfort, and anxiety/depression), the impact of disease activity level on EQ-5D-5L may be less direct than on other conceptually simpler outcome measures.

For proper interpretation of this study results, it should be noted that the result on productivity loss in this study is likely to be conservative for the following reasons. First, the majority of the patients in the claims database were survey non-responders who exhibited higher levels of comorbidity, direct medical cost and HCRU than survey responders whose responses were used to quantify productivity loss. Second, potential productivity loss from caregivers^13,53^ was not considered because it was not part of the standard version of WPAI questionnaire used for this study. Third, analysis of a medical opinion letter for injury and illness benefits suggests presence of severe absenteeism among some patients in the past not captured by the WPAI responses. Thus, economic and humanistic burden of hematologic malignancies at the societal level in the real-world settings may be even greater than those reported by survey responders in this study.

This study does have several limitations. Because of the employment-based nature of the JMDC database, there was under-representation of patients above 65 years old. This population was drawn from an employer-based health claims database and thus is, by definition, comprised of employed individuals and their dependents, and is therefore healthier and more digitally literate than the general Japanese population. Our findings, particularly those related to productivity loss, are generalizable to this insured, working-age population but not to the Japanese population as a whole. From our perspective based on the objective of this study, it has limited impact on the conclusions of this study which set out to focus on patients in the working age population and analyze the magnitude of their productivity loss. Certain information within the JMDC database such as timing of survey was available only at the granularity of monthly units rather than dates, enabling temporal identification and assessment of such events only at monthly levels. While all data were collected retrospectively during the COVID-19 period, our analysis showed that only 4 (3.3%) of 122 patients in this study who responded to survey showed confirmed COVID-19 diagnosis. Thus, in combination with the precedence shown by a previous research in Japan^7^, it is likely that COVID-19 impact was limited in this study. The look-back period during which direct cost outcomes, HCRU, and comorbidities were measured in this study had to be limited to 3 months of continuous enrollment to obtain a sample size adequate to support data analysis; subsequent annualization of cost estimates for ease of interpretation involved assumption of constant utilization rate throughout a year^37^. For future work, a greater sample size is desirable to enable a longer look-back period which can more accurately account for changing utilization rates throughout a year. Direct costs were estimated only for items for which the number of reimbursement points or amount of money could be ascertained, and non-reimbursed items such as usage of over the counter (OTC) medications or traditional herbal medicine were not included because of absence of such data within the insurance claims database. The productivity loss calculation method used in this study does not take account of caregiver burden. Quantification of caregiver burden can be considered in a future study explicitly designed to recruit and elicit PRO from caregivers of patients with hematologic malignancies. Furthermore, our analyses are based on responders who completed the survey. This group may not be representative of all Pep-up users with the same diagnosis. This potential responder bias means our findings may be influenced by unmeasured differences in health-seeking behavior, health literacy, or disease severity between those who did and did not participate. The results should be interpreted with this limitation in mind. In order to go beyond the retrospective analysis of only standardized PRO instruments for general purposes, a prospective follow-up research is desirable in order to collect further data customized for an expanded scope of research and to elucidate the potential reasons for various observations in this study, such as a higher non-working rate among female patients, a relatively high level of QoL among the non-working survey responders and the disease activity level having more impact on cost outcomes than QoL. We did not analyze outcomes stratified by specific treatment modality (e.g., route of administration, targeted vs. cytotoxic) or treatment setting (inpatient vs. outpatient). These categories are complex and often represent poor proxies for the true clinical and toxicity burden. Future research, specifically designed and powered to compare the QOL impact of specific treatment regimens, is necessary to parse these important, heterogeneous patient experiences. Our methodology cannot capture the productivity loss from the cohort of patients who had already permanently left the workforce due to their illness before the study period began. Our data is, therefore, limited to those who were still actively employed at the time of the survey. Consequently, the indirect cost and productivity loss figures presented in this paper should be interpreted as a conservative estimate that likely under-represents the true, total societal burden of this disease. While future research will address these limitations, the current exclusion of chronic cancers such as Chronic Myeloid Leukemia (CML) and Myeloproliferative Neoplasms (MPN) limits the scope of our findings concerning chronic disease management. Consequently, productivity loss estimates may be skewed toward acute morbidity and hospitalization rather than long-term presenteeism, a pattern evident in the cost distribution. Moreover, the divergence between our findings and prior literature^54^ may reflect the inclusion of low-cost observation phases in our longitudinal data. Within this framework, the mean illustrates the total societal budget impact driven by the minority of patients undergoing aggressive interventions, while the median offers a superior proxy for the typical monthly burden during stable disease phases driven by the majority of patients monitored as outpatients. While this study provides a broad cohort overview, future research should utilize larger, multi-institutional datasets to achieve the statistical power necessary for granular, disease-specific analyses. This would enable more precise comparisons of healthcare costs and productivity loss across individual subtypes.

Relevant evidence from this study should be evaluated in conjunction with proper considerations given to the sustainability of healthcare system with upcoming innovative therapies^55,56^ as well as relative cost effectiveness profiles of listed therapies as standard of care^57^. Loss in productivity and QoL suffered by patients of hematologic malignancies who must continue to work while receiving treatment at hospitals invites consideration for what should be an appropriate policy from the perspective of social security in Japan. The need for social sustainability of the healthcare system should be balanced with the additional societal benefits such as increase in productivity, tax revenue, and QoL that further governmental-level support to those patients and their caregivers may generate through better workplace support programs and caregiver resources.

## Conclusion

This study has shown that productivity loss among patients with hematologic malignancies in Japan was sizable among survey responders and greater in magnitude than their direct medical cost. In combination with reduction in QoL among those patients and the conservative nature of the study findings, our analysis demonstrates the presence of considerable economic and humanistic burden for patients of hematologic malignancies who have been treated with non-curative therapies. Innovative therapies with curative potential may be able to address such unmet needs, delivering more social value in the real-world settings than can be inferred based on analysis of direct medical cost alone.

## Supplementary Information

Below is the link to the electronic supplementary material.

Supplementary Material 1

## Data Availability

The datasets generated and/or analyzed during the present study are not publicly available because they were purchased from commercial providers (JMDC Inc.) but are available from the corresponding author upon reasonable request.
